# Effect of hip fracture on prognosis of acute cerebral infarction

**DOI:** 10.6061/clinics/2021/e3059

**Published:** 2021-12-01

**Authors:** Jiawen Yuan, Gang Zhu, Yuwu Zhao, Jiankang Huang

**Affiliations:** I Department of Neurology, Shanghai Jiaotong University Affiliated Sixth People's Hospital, Shanghai 200233, China.; II Department of Neurology, Shanghai Putuo District Central Hospital, Shanghai 200062, China.; III Department of Neurology, Shanghai Jiaotong University Affiliated Sixth People's Hospital, Shanghai 200233, China.; IV Department of Neurology, Nanjing Lishui People's Hospital, Zhongda Hospital Lishui Branch, Southeastern University, Nanjing 211200, China.

**Keywords:** Hip Fracture, Acute Ischemic Stroke, Prognosis

## Abstract

**OBJECTIVES:**

Hip fractures are a worldwide public health problem. The incidence of hip fracture is high among the elderly, and it is an important cause of death and disability in this population. This observational study aimed to investigate the effect of acute hip fracture on the recovery of neurological function and the prognosis of patients with acute cerebral infarction, as well as whether surgical treatment of combined acute fracture can improve the prognosis of patients.

**METHODS:**

Thirty patients with acute cerebral infarction combined with acute hip fracture, who were hospitalized in two hospitals between January 1, 2013 and December 31, 2019, were included. The patients did not undergo surgical treatment. The control group included patients with common acute cerebral infarction without hip fracture admitted in the same period. The neurological function recovery, hospitalization period, half a year recovery rate, incidence of complications, and one-year mortality rate between the two groups were compared. Eleven patients with acute cerebral infarction combined with hip fracture, who underwent surgical treatment, were selected and compared with those in the non-surgery group.

**RESULTS:**

Compared with patients with common acute cerebral infarction, the National Institutes of Health Stroke Scale score of those with acute cerebral infarction combined with hip fracture was higher (7.2±5.4 *vs*. 5.6%±4.3, *p*=0.034), the hospitalization period was prolonged (16.1±8.9% *vs*. 12.2±5.3, *p=*0.041), and the half a year recovery rate was lower (26.7% *vs*. 53.3%, *p*=0.016). Additionally, the incidence of pulmonary infection and lower extremity deep vein thrombosis was increased (30% *vs*. 11.7%, *p*=0.03; 6.7% *vs*. 0, *p*=0.043). The one-year mortality rate of patients with hip fracture was higher than that of patients with common cerebral infarction (23.3% *vs*. 6.7%, *p*=0.027). Compared with the non-surgical group, the good recovery rate after half a year of surgical treatment of the group with cerebral infarction and acute hip fracture had an increasing trend, while the hospitalization cycle, incidence of complications, and one-year mortality rate were all decreased, although this was not statistically significant.

**CONCLUSIONS:**

Acute cerebral infarction combined with hip fracture leads to worse neurological recovery, prolonged hospitalization period, increased complications, decreased patient prognosis, and increased one-year mortality. Surgical treatment improves the prognosis of patients with acute cerebral infarction. These findings may provide insights into the management of acute cerebral infarction.

## INTRODUCTION

Hip fractures are a worldwide public health problem with a high incidence among the elderly and an important cause of death and disability in this population. The prognosis of patients with hip fracture is poor. Many patients cannot recover to their functional state before fracture. Nearly 40% of patients cannot walk independently after discharge (1). Nearly 25% of the patients need long-term care, and 20-30% of them die within one year after the onset of the disease (2). Acute stroke is the leading cause of death and disability worldwide (3), among which acute ischemic stroke accounts for approximately 70%. Acute stroke is an important risk factor for hip fracture. Clinical studies have shown that acute stroke in elderly patients is closely related to hip fracture, and the risk of falls is significantly increased because of balance dysfunction and sensory impairment. The risk of hip fracture after stroke is 1.5-7 times higher than that of general patients (4-6). Ramnemark et al. studied 1139 patients with acute stroke and found that the incidence of hip fracture was 2-4 times higher than that of the general population reported in the literature (7). Cerebral infarction and fracture have the same risk factors; therefore, patients who are prone to cerebral infarction are also prone to bone loosening and fracture. The risk factors for post-stroke fracture include osteoporosis, aging, and postural instability. Osteoporosis is the most important risk factor for osteoporosis. Fracture after stroke makes patients at a greater disadvantage in the rehabilitation process and greatly affects the quality of life after rehabilitation. In clinical observation, we also found that fracture after stroke prolonged the hospitalization period of patients and increased the incidence of complications such as pulmonary infection and venous thrombosis, which may increase the risk of death of patients. For patients with hip fracture after acute cerebral infarction, orthopedic doctors believe that acute cerebral infarction is a relative contraindication for surgery; therefore, most of these patients cannot undergo surgical treatment. However, there are several patients requesting surgical treatment because family members insist on fracture internal fixation or hip replacement. Whether surgery is conducive to the recovery of patient conditions and improves prognoses is not well studied, as there are not many clinical reports on this topic and most existing research is based on animal models. We retrospectively analyzed patients with hip fracture after acute cerebral infarction and discussed the influence of hip fracture on hospitalization period, neurological function recovery, complication rate, and one-year mortality rate of patients with acute cerebral infarction, as well as the influence of surgical treatment of acute hip fracture on patient prognosis.

## MATERIALS AND METHODS

### General information

In this observational study, patients with acute cerebral infarction complicated with hip fracture, who were hospitalized in the neurology, orthopedics, and emergency wards of the two hospitals between January 1, 2013 and December 31, 2019, were screened using the electronic medical record system of Shanghai Jiaotong University Affiliated Sixth People’s Hospital and Shanghai Putuo District Central Hospital. The patients’ hip fracture occurred within 3 days after acute cerebral infarction. Patients with prior cerebral infarction and those with acute cerebral infarction after hip fracture were excluded. Patients with pathological fracture were excluded from this study. A total of 41 patients with acute cerebral infarction complicated with hip fracture were screened, including 11 who undergo surgery in the acute stage and 30 who did not undergo surgery. A total of 60 patients with age-and sex-matched acute cerebral infarction were included in the control group. Ethical approval for the study was granted by the Medical Ethics Committee of Shanghai Jiaotong University Affiliated Sixth People’s Hospital, in Shanghai, China.

### Methods

Data on the demographic characteristics, basic diseases (such as hypertension, diabetes, and atrial fibrillation history), main diseases, blood routine, blood biochemistry, brain magnetic resonance imaging or computed tomography scans, and complications during hospitalization (such as deep vein thrombosis and pulmonary infection) of all selected patients were obtained through the electronic medical record system. According to the statistics of neurological function and mortality after one year of telephone follow-up, the prognosis of patients whose Modified Rankin Scale (mRS) score was <3 was considered good.

### Statistical methods

SPSS 20 (IBM SPSS, USA) was used for statistical analyses of the measurement data, and the measurement data between two groups were expressed as x̅±s. The comparison of measurement data between the two groups was conducted using the *t*-test. The positive rate between the groups was compared using the chi-square test. There was a significant difference between the two groups (*p<*0.05).

## RESULTS

The basic information of 30 patients with acute cerebral infarction combined with hip fracture who did not undergo surgical treatment was recorded, including 15 male patients accounting for 50% of the cohort, with an average age of 81.3±8.4 years. There were 60 patients in the control group, including 32 male individuals, accounting for 53.3% of the cohort, with an average age of 79.6±9.5 years. The incidence of basic diseases and complications between the two groups are shown in Table 1. There was no significant difference in the incidence of basic diseases, including hypertension, diabetes, chronic kidney disease, and atrial fibrillation, between the two groups (Table 1).

Acute cerebral infarction combined with hip fracture affected the recovery of neurological function and prolonged the hospitalization period. The National Institutes of Health Stroke Scale (NIHSS) score of patients with acute cerebral infarction was 8.2±5.2 at admission, and that of patients with hip fracture was 8.7±6.3; there was no significant difference between the two groups. The NIHSS score of patients with hip fracture was 7.2±5.4 at discharge, and the recovery was worse than that of patients with common acute cerebral infarction (*p*=0.034). The average hospitalization period of patients with hip fracture was significantly longer than that of patients with common acute cerebral infarction (16.1±8.9 *vs*. 12.2±5.3, *p*=0.041). The recovery rate of patients with hip fracture was significantly lower than that of patients with common cerebral infarction (53.3% *vs*. 26.7%) (Table 2).

Patients with acute hip fracture had a high incidence of complications and increased mortality. The incidences of pulmonary infection and deep venous thrombosis of the lower limbs in patients with acute cerebral infarction and hip fracture were analyzed. The incidence of pulmonary infection in patients with hip fracture was 30%, while that in patients with common acute cerebral infarction was 11.7%. There was a significant difference between the two groups. There were no cases of deep vein thrombosis in the common acute cerebral infarction group, and the difference was statistically significant. The one-year mortality of patients with hip fracture was significantly higher than that of patients with common cerebral infarction (23.3% *vs*. 6.7%, *p*=0.027) (Table 3).

Patients with acute hip fracture recovered well after surgery. We enrolled 11 patients with hip fracture after acute cerebral infarction. The average NIHSS score of this group upon admission was 8.4±4.3, and that at discharge was 7.3±4.4. There were no significant differences between the two groups. Five patients recovered well after half a year (mRS score ≤3), accounting for 45.5%, while 26.7% of patients with hip fracture did not receive surgical treatment. The rate of good recovery of the surgery group was significantly higher than that of the non-surgery group, although the difference was not statistically significant. The hospitalization period was 14.1±6.2 days, there was one case of pulmonary infection (11.1%), no patients developed deep venous thrombosis of the lower extremities, and one patient died within one year (11.1%), all of which were much lower than the values of the non-surgery group; however, there was no significant differences between the two groups. The sample size was too small, which may have led to the lack of significant differences.

## DISCUSSION

Hip fracture is a common type of fracture in the elderly. A large-scale study in China showed that hip fracture is the second-most common type of fracture, followed by distal radius fracture (8). Hip fracture seriously affects the limb function and quality of life of patients, known as the “last fracture.” A single-center retrospective study showed that the mortality rate of patients with hip fracture in the non-surgical group was four times higher than that in the surgical treatment group within one year and three times higher within two years (9). Another follow-up study showed that among non-surgical patients, the 30-day mortality rate of long-term bedridden patients was 3.8 times higher than that of patients in the early activity group (10). The study also showed that there was no significant difference in mortality between surgical and non-surgical but early-active patients. Early recovery is very important for patients with hip fracture. The data suggest that pain, bleeding, and inactivity associated with acute hip fractures can lead to inflammation, hypercoagulability, and catabolism, all of which may have adverse consequences. International guidelines recommend hip fracture surgery within 48h of the event. This recommendation is based on the results of an observational study that showed that a shorter operation time can improve the prognosis of patients (11). Clinical observations have shown that acute cerebral infarction combined with acute hip fracture significantly affects the recovery of neurological function and prognosis; these cases are also more prone to complications. This retrospective analysis showed that patients with acute hip fracture had higher NIHSS scores and longer hospitalization periods than patients with common cerebral infarction, suggesting that their recovery of neurological function was worse. The rate of good prognosis in patients with hip fracture in half a year was significantly lower than that in patients with common cerebral infarction. The incidence of pulmonary infection and deep venous thrombosis in the lower limbs was significantly increased. The one-year mortality rate of patients with hip fracture was significantly higher than that of patients with common cerebral infarction. Degos et al. found that the occurrence of fracture one day after acute cerebral infarction could aggravate neuronal damage and dysfunction, which is related to the aggravation of neuroinflammation and oxidative stress (12). The results of surgical treatment for patients with cerebral infarction complicated with hip fracture have been rarely reported, and the conclusions are inconsistent. One study reported that the mortality rate of patients with hip fracture with a history of stroke was higher than that of patients without a history of stroke (13). A study from South Korea reviewed 548 patients who underwent hip fracture surgery within 5 years in their hospital. Among them, 77 patients had a history of stroke. There was no significant difference in the one-year mortality between the two groups (14). Currently, there are no case studies on the surgical treatment of hip fracture after acute cerebral infarction. In our collected cases, the overall treatment effect in patients with acute cerebral infarction complicated with acute hip fracture was good, including good prognosis rate, hospitalization period, pulmonary infection, and deep vein thrombosis complications, although there were no statistical differences between the two groups. Whether hip fracture surgery can benefit patients with acute cerebral infarction remains to be further clarified in clinical studies with larger sample sizes.

## CONCLUSION

Acute cerebral infarction combined with hip fracture leads to worse neurological function recovery, longer hospitalization period, more complications, lower good prognosis rate, and higher one-year mortality rate. Surgical treatment of hip fracture may improve the prognosis of these patients, which requires further study.

## AUTHOR CONTRIBUTIONS

Yuan J and Huang J were responsible for the conception and design of the manuscript. Yuan J, Zhu G and Huang J were responsible for the acquisition, analysis, and interpretation of data. Zhao Y and Huang J were responsible for the drafting of the manuscript and for its critically review for important intellectual content. All of the authors approved the manuscript final version to be published.

## Figures and Tables

**Table 1:** Demographic data and basic diseases of patients.

	Acute cerebral infarction (n=60)	Acute cerebral infarction combined hip fracture (n=30)	*p*-value
Gender (male)	32 (53.3%)	15 (50%)	0.765
Age (years)	79.6±9.5	81.3±8.4	0.653
Hypertension	33 (55%)	18 (60%)	0.652
Diabetes	20 (33.3%)	9 (30%)	0.750
Atrial fibrillation	11 (18.3%)	6 (20%)	0.849

**Table 2:** Neurological function and hospitalization period of patients.

	Acute cerebral infarction patients (n=60)	Acute cerebral infarction combined with hip fracture (n=30)	*p*-value
NIHSS admission	8.2±5.2	8.7±6.3	0.606
NIHSS discharged	5.6±4.3	7.2±5.4	0.034
Hospitalization period (days)	12.2±5.3	16.1±8.9	0.041
Half-year recovery rate	32 (53.3%)	8 (26.7%)	0.016

**Table 3:** Complications and mortality.

	Acute cerebral infarction patients (n=60)	Acute cerebral infarction combined hip fracture (n=30)	*p*-value
Pulmonary infection	7 (11.7%)	9 (30%)	0.032
Lower limbs deep venous thrombosis	0	2 (6.7%)	0.043
One-year mortality	4 (6.7%)	7 (23.3%)	0.027
